# Extract from the Minutes of a Convention of Surgeon Dentists, Assembled in the City of Richmond, Va

**Published:** 1842-12

**Authors:** S. Lethbridge, James D. McCabe

**Affiliations:** President.; Secretary.


*Extract from the Minutes of a Convention of Surgeon Dentists, assembled in the City of Richmond, Va. on Monday, December 12th, 1842.*

At half past 3 o'clock, the Convention was called to order by Dr. James D. McCabe, who nominated Dr. S. Lethbridge, of Richmond, to the chair, and on motion of Dr. John McConnell, Dr. Jas. D. McCabe was appointed secretary.

The following gentlemen were enrolled as present in person or by proxy. S. Lethbridge, J. D. McCabe, R. N. Hudson, John McConnell, John G. Wayt, Richmond; S. M. Sheppard, Dr. Murrell, Dr. Sheppard, Petersburg; T. B. Hamlin, Wyth C. H. Va.; D. M. Coulling, King and Queen Co. Va.; Wm. M. McKenney, J. B. Garland, Fredericksburg; W. W. H. Thackston, Farmville; W. C. Crump, Washington, N. C.; C. F. Martin, Norfolk; J. C. Birch, Charlottesville; A. Pierce, Sussex C. H.; J. C. McCabe, Richmond, Va.

On motion, resolved, That in the opinion of this Convention, the dental science would be greatly benefitted and advanced, and the interests of its practitioners, and of the community at large, promoted by the formation of a state society, for the purpose of uniting the energies of the profession, to distinguish and reward merit—to discountenance and expose quackery and charlatanism; therefore, be it further resolved, that a committee be appointed to report to this Convention a form of constitution for the organization and government of such a society.

A form of constitution prepared by Dr. W. W. H. Thackston, was reported to the convention, which, after slight modification was adopted as the constitution of the "Virginia Society of Surgeon Dentists."*

The convention then proceeded to organize under the constitution by the election of the following officers to serve for the next twelve months.

Dr. S. Lethbridge, *President;* Dr. John G. Wayt, *Vice-president;* Dr. James D. McCabe, *Corresponding and Recording Secretary;* Dr. S. M. Sheppard, *Treasurer;* Dr. W. W. H. Thackston, Dr. R. N. Hudson, Dr. John McConnell, Dr. T. B. Hamblin, Dr. William M. McKenney, *Executive, Examining and Publishing Committee.*

On motion, Dr. James D. McCabe was requested to deliver the address at the first annual meeting of the society.

On motion, ordered that the following scale of prices be adopted by this society, and recommended to the profession in Virginia as the minimum and maximum of dental charges.

| | | | | |
|---|---|---|---|---|
| For plugging | from | $1 00 | to | $5 00 |
| For extracting | do. | —— | to | 1 00 |
| For separating | do. | 50 | to | 1 00 |
| For scaling | do. | 2 00 | to | 5 00 |
| For inserting on pivot | do. | 4 00 | to | 7 00 |
| For inserting on plate | do. | 7 00 | to | 12 00 |
| Full sets | do. | 150 00 | to | 300 00 |

The society then proceeded to consider and adopt a code of by-laws for its government.

On motion, resolved, that Drs. James D. McCabe, John G. Wayt and S. M. Sheppard, be, and are hereby constituted a committee to apply to the legislature of Virginia for an act of incorporation for this society.

On motion, the President was added to the committee.

The following gentlemen were appointed to prepare essays to be read at the annual meeting, leaving it to themselves to select the subjects.

Dr. D. M. Coulling, Dr. R. N. Hudson, Dr. John M. Connell, Dr. W. W. H. Thackston, Dr. W. W. McKenney.

On motion, resolved, that the committee appointed to procure a charter from the legislature be instructed to obtain a proper seal for the use of this society.

On motion, resolved, that the first annual meeting of this society be held in the city of Richmond on the second Wednesday in October next.

On motion, ordered, that the secretary publish the proceedings of this meeting, together with the constitution and by-laws, and that he furnish each member with six copies.

On motion, ordered, that a committee of three be appointed to prepare an address to the dental profession in Virginia.

* The constitution and by-laws are those of the American Society of Dental Surgeons, modified to suit the circumstances of locality.

The chair named the committee to consist of Drs. McCabe, Wayt and Hudson.

On motion, ordered, that this society now adjourn to the second Wednesday in October next, to meet in the city of Richmond.

S. LETHBRIDGE, *President.*

James D. McCabe, *Secretary.*

